# Implementation of the Finnish Good Practice “Smart Family” in Poland

**DOI:** 10.3390/children12111437

**Published:** 2025-10-23

**Authors:** Justyna Nowak, Agata Szymczak, Barbara Kaczmarska, Katarzyna Anna Klonowska, Marta Morawska, Heli Kuusipalo, Emma Koivurinta, Kati Kuisma, Päivi Mäki, Taina Sainio, Nella Savolainen, Katarzyna Brukało

**Affiliations:** 1 Department of Cardiovascular Disease Prevention, Department of Metabolic Disease Prevention, Faculty of Public Health in Bytom, Medical University of Silesia, 41-900 Bytom, Poland; 2 Office of Public Partnership and Innovation, National Health Fund, Central Office, 02-528 Warsaw, Poland; 3 Department of Healthcare Services, National Health Fund, Central Office, 02-528 Warsaw, Poland; 4 Department of Analysis, Quality Monitoring and Optimization of Benefits, National Health Fund, Central Office, 00-863 Warsaw, Poland; 5 Finnish Institute for Health and Welfare, 00271 Helsinki, Finland; 6 Finnish Heart Association, 00621 Helsinki, Finland; 7 Department of Health Policy, Faculty of Public Health in Bytom, Medical University of Silesia, 41-902 Bytom, Poland

**Keywords:** childhood obesity, family-centered intervention, Smart Family method, health promotion

## Abstract

**Background:** Childhood obesity is a growing public health challenge in Poland and worldwide, associated with serious long-term health consequences. Effective prevention requires family-centered, evidence-based interventions that actively engage both children and their caregivers. This study presents the Finnish Smart Family practice—an evidence-based lifestyle counseling method developed by the Finnish Heart Association—and describes its adaptation and implementation in Poland as part of the EU Health4EUkids project. The study emphasizes the method’s practical utility for professionals working with families of children with obesity. **Methods:** The Smart Family approach is a structured lifestyle counseling method based on findings from the Special Turku Coronary Risk Factor Intervention Project (STRIP) that is grounded in health psychology and strength-based counseling principles. Unlike traditional counseling, which focuses mainly on information transfer, Smart Family promotes motivation, families’ active participation, and recognition of their strengths in areas such as nutrition, physical activity, sleep, and oral hygiene. The method uses practical tools including the Smart Family card, other supporting materials, and dedicated online platforms for both families and healthcare providers. These tools enable families to self-assess their lifestyle, select discussion topics during visits, and set achievable goals while supporting professionals in initiating non-judgmental, collaborative conversations. In Poland, the program was adapted using culturally appropriate materials and professional training, followed by pilot implementation in primary healthcare and educational settings that included pre-implementation planning, practical training sessions, the application of intervention tools, and outcome evaluation. **Results:** Pilot implementation demonstrated high usability and effectiveness. The approach enabled non-judgmental, supportive engagement with families, facilitated active participation in setting health goals, and promoted sustainable lifestyle changes in nutrition, physical activity, sleep, and other health behaviors. Evaluation highlighted the importance of supporting program objectives at the national level, standardizing child healthcare practices, and engaging media and local authorities to create a supportive ecosystem. **Conclusions:** The Polish experience confirms that Smart Family is an evidence-based intervention that strengthens professional competence, provides practical tools for family-centered care, and supports the long-term prevention of child-hood obesity and related non-communicable diseases. Its integration into healthcare and educational settings offers a promising strategy for improving public health outcomes.

## 1. Introduction

Between 1990 and 2021, the prevalence of overweight and obesity among children and adolescents increased dramatically, with the combined rate doubling and obesity alone tripling. By 2021, approximately 93 million children aged 5–14 years and 81 million young people aged 15–24 years were affected by obesity. Although the prevalence of overweight (excluding obesity) is expected to stabilize between 2022 and 2050, the absolute number of individuals with obesity is projected to rise even more sharply than in the previous decades, particularly between 2022 and 2030. This anticipated surge underscores the urgent need for immediate public health interventions [1].

Obesity and overweight currently affect approximately 10% of children and adolescents worldwide. By 2025, it is projected that 177 million children aged 5–17 years will be overweight including 91 million who will meet the criteria for obesity. According to the World Health Organization (WHO), obesity currently affects, on average, 1/3 boys and 1/5 girls aged six to nine years. This rapid rise in childhood obesity is accompanied by a corresponding increase in obesity-related comorbidities. Based on current trends, it is estimated that by 2025, approximately 12 million children will exhibit abnormal glucose tolerance, 4 million will develop type 2 diabetes, 27 million will suffer from hypertension, and 38 million will have hepatic steatosis or fat accumulation in the liver. These alarming projections emphasize the necessity of adopting effective preventive strategies and early interventions to reduce the long-term health consequences of childhood obesity [2].

In Poland, the prevalence of overweight and obesity among children and adolescents is a growing public health concern. According to WHO data, 12.2% of boys and 10% of preschool girls are affected by overweight or obesity. Estimates suggest that more than 20% of children of primary school age are overweight, and a pre-COVID-19 report commissioned by the Ministry of Health indicated that the prevalence reaches 30.5% among children of primary school age in Poland, well above the EU average of approximately 19%. In primary and secondary school, the prevalence rises to 18.5% among boys and 14.3% among girls. Regionally, the highest proportions of overweight and obese children are observed in the Mazowieckie, Lubuskie, Lower Silesia, and Kujawsko-Pomorskie voivodeships, while the lowest rates are reported in Małopolska, Świętokrzyskie, Lubelskie, and Podkarpackie [3]. These alarming figures underline the urgent need for preventive strategies, early interventions, and targeted public health policies to address the rising burden of childhood obesity in the country.

The factors contributing to childhood obesity are complex, and no single intervention is sufficient to prevent it. Effective prevention requires multi-level, sustained actions involving schools, communities, governments, private organizations, and non-governmental organizations (NGOs). Interventions should start early—from pregnancy through childhood—and address all relevant risk factors for non-communicable diseases including unhealthy diet, low physical activity, tobacco, and alcohol use. Integrating obesity prevention into broader strategies for nutrition, physical activity, and early childhood development is essential, as isolated measures alone are unlikely to produce a significant impact. Schools and community networks, supported by governmental and non-governmental organizations, can play a key role by promoting healthy behaviors within obesogenic environments [4,5].

In Poland, several initiatives have been carried out to reduce overweight and obesity among children and adolescents, following the recommendations of the World Health Organization and the European Strategy on Nutrition, Physical Activity, and Health. Key programs included: Junior-Edu-Nutrition (JEŻ)—a nutrition education program for students in grades 1 to 6 in primary schools, conducted by teachers and experts in dietetics and nutrition; DINO-PL (Diagnosis–Intervention–Hypertension–Obesity)—a program for monitoring, early diagnosis, and intervention regarding overweight, obesity, and high blood pressure in children aged 7 to 15 years; the Obesity Treatment Program at the Institute of Mother and Child, providing comprehensive, multi-specialist care for children with obesity; and “Healthy and Active”, run by the sports company Decathlon and the Decathlon Foundation, promoting physical activity among preschool and early school-age children. Additionally, in primary healthcare, coordinated care by family doctors (primary care physicians) was implemented, including assessment, nutrition education, and support for physical activity. All these initiatives were multi-level, involving the home, school, and local community environments, and aimed both at preventing and the early treatment of overweight and obesity among children [6,7,8,9,10].

The aim of this study is to present the Smart Family method, a Finnish lifestyle counseling practice, and to describe its implementation in the Polish context. The study also aims to demonstrate how this evidence-based practice, recognized by the European Commission as a best practice, contributes to the promotion of healthy lifestyles and the prevention of childhood obesity and other non-communicable diseases.

## 2. Materials and Methods

Health4EUkids is an European Union project aimed at spreading best practices in health promotion and the prevention of non-communicable diseases across several European countries, with a focus on childhood obesity. The project implements two facilitated methods: Grunau Moves from Germany and the Finnish Smart Family method, while sharing knowledge and experience between the owners of these practices and partner organizations in the member states. The implementation includes practical activities, cooperation and knowledge exchange between countries, organizing meetings, and providing technical support. An important part of the project is also creating policy recommendations to support public health investments at the local and national levels, promoting healthy lifestyles in families with children, increasing physical activity, encouraging healthy diets, and supporting social awareness and self-esteem, especially in disadvantaged areas. The project also studies the conditions in each country, ensures the sustainability of implemented practices, and prepares them for transfer to other member states based on the experience gained. Health4EUkids aims to help develop policies and strategies at the European, national, regional, and local levels that effectively prevent childhood obesity [11].

The Smart Family method (Neuvokas perhe), developed in Finland by the Finnish Heart Association, is a comprehensive lifestyle counseling practice designed to support families in building healthy habits and preventing childhood obesity. Unlike traditional approaches that focus only on providing information, Smart Family emphasizes active engagement, motivation, and encouragement, helping families identify their strengths in nutrition, physical activity, sleep, oral hygiene, and other health-related behaviors. The method uses practical tools such as the Smart Family card, a picture folder for professionals, and dedicated websites for both families and healthcare providers [12,13]. These tools enable families to self-assess their lifestyle, choose discussion topics during healthcare visits, and actively participate in setting goals, while professionals are supported in opening non-judgmental conversations and guiding behavior changes. Since its launch in 2008, over 5000 public health nurses and professionals in Finland have been trained to use the method, and more than 370,000 Smart Family cards have been distributed. The program has shown high satisfaction rates among both families and professionals, and its effectiveness has been confirmed by research demonstrating increased family empowerment, self-efficacy, and the recognition of existing strengths in maintaining a healthy lifestyle. Importantly, Smart Family is fully integrated into routine healthcare work, making it a sustainable and scalable best practice recognized at the European level [12,13,14].

Since December 2022, Poland has been one of six countries implementing the Finnish Smart Family practice as part of the Joint Action Health4EUkids. In Poland, the project is coordinated by the National Health Fund, with the Medical University of Silesia in Katowice acting as an Affiliated Entity. Alongside Poland, the Smart Family method, recognized by the European Commission as a best practice, is also being implemented in Greece, Slovenia, Croatia, Lithuania, and the Balearic Islands of Spain [11,12,13].

### Implementation Process, Participant Description, Adaptation, and Evaluation

The Smart Family program was introduced in Poland in November 2023 to support families of children with excess body weight. The initiative primarily targeted professionals working with these families including dietitians, nurses, and school staff such as teachers, counselors, and psychologists. Participants were purposively selected based on inclusion criteria such as final-year nursing or dietetics students, formally educated dietitians, attendees of conferences organized by the Polish Society of Dietetics, or professionals working as teachers, school counselors, or psychologists. All participants provided written informed consent.

The training was conducted in-person following a standardized agenda to ensure consistency across sessions. It combined theoretical lectures with practical exercises and interactive discussions. Key topics included the Finnish Smart Family methodology, principles of positive child development, and strategies to support lifestyle changes. Participants were introduced to motivational interviewing, the transtheoretical model of behavior change, and structured goal-setting methods using the GROW and SMART frameworks. Central to the training was the use of Smart Family tools including the Healthy Family Card, Star of Our Family, Family Screen Time Agreement, Sleep Tree, etc. All materials were translated into Polish and culturally adapted to ensure relevance for local families and professionals.

Training effectiveness was assessed using a pre-post design. Participants completed questionnaires before and after the sessions to evaluate their knowledge of the Smart Family method, familiarity with the tools for working with families of children with excess body weight, confidence in applying motivational interviewing, and motivation to implement the Smart Family approach in practice. The Implementation Motivation Questionnaire additionally assessed potential barriers and practical skills gained. These combined measures provided a comprehensive assessment of both the usability and effectiveness for professionals.

As an additional implementation step, Smart Family tools were also used by nurses in primary healthcare settings while providing services to the families of children with excess body weight.

## 3. Results and Discussion

### Implementation of the Smart Family Practice in Poland

The Smart Family practice was implemented in Poland based on the Guideline on Implementation Strategy [15].

The first step in implementing the Smart Family method in the Polish healthcare system was to consider the long-term health risks of obesity, which over time lead to higher rates of illness and increased healthcare costs. The growing number of children with overweight and obesity is linked to more complications and related diseases, which puts a greater burden on both patients and the healthcare system. In implementing the method, special emphasis was placed on strengthening the role of pediatricians, family doctors, nurses, school nurses, dietitians, and kindergarten staff—professionals who have the most frequent and long-term contact with children and their parents or caregivers. These relationships make it possible to monitor changes in children’s health, detect early signs of overweight and obesity, and actively involve entire families in promoting a healthy lifestyle. Smart Family interventions are aimed not only at children, but at whole families. By making lasting lifestyle changes at the family level, it is possible to establish and maintain healthy habits related to nutrition, physical activity, sleep, and other health behaviors, which is key to effective prevention of obesity in the Polish social and healthcare context.

The specific aims of implementing the Smart Family method in Poland were focused on three main objectives. First, to develop a model for supporting families with a child who has a non-normative body weight, integrating the role of primary healthcare and environmental health determinants. Second, to increase public awareness of nutrition and encourage health-promoting changes in dietary behaviors. Third, to enhance the competence of primary care staff, teachers, and educators in promoting health and preventing diet-related diseases through a “Train the Trainers” approach.

**Figure 1:**
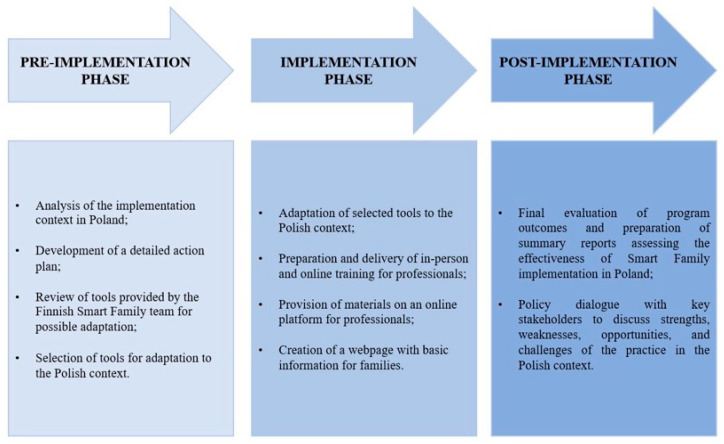
Implementation Process of the Smart Family Practice in Poland: Pre-Implementation, Implementation, and Post-Implementation Phases.

The implementation of the Smart Family practice in Poland was divided into three main phases (Figure 1).

The pre-implementation phase (months 1–13) focused on a detailed analysis of the context and the preparation of an action plan, which was to be completed by the end of November 2023. During this phase, the tools provided by the Finnish Smart Family team were analyzed, and those selected for adaptation to Polish conditions were identified.

The implementation phase (months 14–30) involved putting the program into practice. This included the creation of a project website, presenting an overview of the program, describing the tools, and providing support for both professionals and parents of children [16]. Based on the Finnish tools [12], 11 instruments were adopted and adapted for use in Poland, including: the Healthy Family Card, “Our Family Star” Card, “Child’s Nutrition Habits Star” Card, “Yum” Card, “Exercise” Card, “Sleep Tree” Card, “Child’s Daily Activity Star” Card, “Click” Card, “My Body” Card, “I Can” Card, and “Screen Time Agreement” Card. Additionally, two new tools were developed: the Family Shopping Card and the Habit Change Agreement Card. All tools are freely available on the Polish project website and can be used by any professional working with families of children with excessive body weight. During this phase, comprehensive training materials for nursing and dietetics students were developed, and a series of in-person trainings was launched in several cities across Poland. A total of 295 professionals participated in the training, including 52.2% dietitians, 34.9% nurses, and 12.9% school staff.

Educational materials were also prepared for primary healthcare (PHC) teams and other professionals, alongside an e-learning course for professionals covering four modules: Smart Family tools, the role of motivation and positive communication with families, nutrition principles for school-aged children, and physical activity, sleep, and leisure time. The e-learning course is freely accessible on the National Health Fund website and can be used by any professional working with children with obesity [17]. Additionally, a leaflet entitled “How to Talk About Childhood Obesity? A Guide for Professionals Working with Families in the Healthy Family Program” has been prepared. This publication is intended for professionals and offers practical guidance on how to work with families with children affected by obesity [18].

Intervention activities were also implemented in selected PHC facilities during this phase. The post-implementation phase (months 31–36) focused on the final evaluation of the program’s effects and the preparation of summary reports to assess the effectiveness of implementing the Smart Family method in the Polish healthcare system. As part of this phase, a policy dialogue was conducted with key stakeholders to discuss the strengths, weaknesses, opportunities, and challenges of implementing the practice in the Polish context.

The evaluation of the pilot implementation indicated that the Smart Family methodology and approach were deemed essential by stakeholders for guiding the practical activities implemented within the intervention. The findings also highlighted the necessity of supporting the implementation of the program’s objectives at the national level to ensure its sustainability and scalability. Furthermore, the evaluation underscored the importance of developing a unified national standard of institutional care for children, encompassing healthcare, the educational and upbringing environment, the family setting, and social care. In addition, the results pointed to the need for systematic support of professionals through the active involvement of the media and local authorities, with the aim of building an integrated environment that promotes and sustains healthy lifestyles.

The project is currently in its closing phase, with completion planned for November 2025. As part of the Policy Dialogue, which took place in May 2025, key stakeholders were identified and invited to participate. These stakeholders have a direct influence on shaping and guiding the development of prevention and health promotion activities and are responsible for determining how to implement them through defining standards, algorithms, substantive guidelines as well as rules for the organization and financing of activities. Key stakeholders included the Ministry of Health, the National Food and Nutrition Institute, national consultants on family medicine, family nursing, and pediatric care, the Maternal and Child Institute, the Polish Children’s Obesity Association, and the Supreme Chamber of Nurses and Midwives.

Based on the conclusions drawn during this dialogue, there are realistic prospects for maintaining and continuing the Smart Family practice in the Polish healthcare system. The support of key stakeholders, the positive evaluation of the tools, and the potential for integrating the method into the routine work of primary healthcare teams suggest that the Smart Family approach can be effectively maintained and further developed at the national level.

In summary, the implementation of the Finnish Good Practice Smart Family in Poland highlights the importance of family-centered approaches in tackling childhood obesity. This methodology, developed by the Finnish Heart Association, emphasizes motivation, empowerment, and the recognition of families’ strengths, moving beyond traditional information-based interventions. Within the framework of the EU Health4EUkids project, the practice was adapted to Polish conditions through the development of culturally appropriate tools, training programs, and educational resources for healthcare and education professionals. The pilot implementation demonstrated that the Smart Family approach was rated as practical and applicable in both clinical and community settings. It enabled professionals to initiate non-judgmental, supportive conversations with families, facilitated active participation in setting health goals, and promoted sustainable changes in nutrition, physical activity, sleep, and other lifestyle behaviors. The evaluation also highlighted the necessity of supporting program objectives at the national level, developing uniform standards of child healthcare across institutional, family, and social environments, and engaging media and local authorities to build a supportive ecosystem for healthy living. The Polish experience confirms that Smart Family is a valuable, evidence-based, and scalable intervention that strengthens the competence of professionals and provides practical, user-friendly tools for working with families of children affected by obesity. Its integration into primary healthcare and educational settings offers a promising pathway for the long-term prevention of childhood obesity and related non-communicable diseases, contributing to improved public health outcomes.

## 4. Conclusions

The Polish experience confirms that Smart Family is an evidence-based intervention that strengthens professional competence, provides practical tools for family-centered care, and supports long-term prevention of childhood obesity and related non-communicable diseases. Its integration into healthcare and educational settings offers a promising strategy for improving public health outcomes.

## Data Availability

The data presented in this study are available on request from the corresponding author. The data are not publicly available due to the large volume of data and to maintain clarity and readability.

## Abbreviations

The following abbreviations are used in this manuscript:

WHO	World Health Organization
NGOs	Non-Governmental Organizations
JEŻ	Junior-Edu-Nutrition
DINO-PL	Diagnosis–Intervention–Hypertension–Obesity
